# Cyclic GMP-mediated intercellular communication in mammalian ovarian follicles

**DOI:** 10.1186/2050-6511-14-S1-P31

**Published:** 2013-08-29

**Authors:** Laurinda A Jaffe, Jeremy R Egbert, Leia C Shuhaibar, Lai Wen, Martin Thunemann, Robert Feil, Viacheslav Nikolaev, Jerid W Robinson, Lincoln R Potter

**Affiliations:** 1 Department of Cell Biology, University of Connecticut Health Center, Farmington CT 06030, USA; 2 Interfakultäres Institut für Biochemie, University of Tübingen, Tübingen 72076, Germany; 3 Department of Cardiology and Pneumology, University of Göttingen, Göttingen 37075, Germany; 4 Department of Biochemistry, Molecular Biology, and Biophysics, University of Minnesota, Minneapolis, MN 55455, USA

## Background

In mammalian ovarian follicles, granulosa cells keep fully grown oocytes arrested in meiotic prophase. A key inhibitory signal is cGMP, which diffuses into the oocyte from the granulosa cells, where it is synthesized by guanylyl cyclase B/ natriuretic peptide receptor 2 (NPR2) in response to the agonist C-type natriuretic peptide (CNP) [1]. Then in response to luteinizing hormone (LH), cGMP in the granulosa cells and oocyte decreases, promoting resumption of meiosis [2]. The primary mechanism by which LH signaling reduces cGMP in the granulosa cells is by reducing the activity of NPR2; this occurs by a rapid modification of the NPR2 protein, followed by a decrease in CNP in the ovary [3].

## Methods and results

Using knock-in mice expressing the cGMP FRET sensor cGi500 [4], we determined that the cGMP decrease begins in the outer layers of granulosa cells where the G-protein-coupled receptors for LH are located [5]. In these cells, the concentration of cGMP decreases to a plateau level with a half time of ~2 min. Because the cells of the follicle are connected by gap junctions [6], cGMP also decreases in the inner layers of the follicle, and finally in the oocyte, where the [cGMP] decreases with a half time of ~10 minutes. This rapid cGMP decrease throughout the follicle correlates with our previous finding that the guanylyl cyclase activity of NPR2 has decreased by ~50% at 20 minutes after applying LH [3].

Because NPR2 activity depends on phosphorylation of several juxtamembrane serines and threonines [7], we investigated whether the LH-induced decrease in NPR2 activity might be due to dephosphorylation. The decrease in guanylyl cyclase activity measured at 20 minutes after LH application was inhibited by preincubating the follicles with the phosphatase inhibitor okadaic acid, suggesting that LH signaling decreases NPR2 activity by dephosphorylating the protein. To determine if dephosphorylation occurred, we immunoprecipitated NPR2 from rat follicle membranes, and separated phosphorylated forms of NPR2 using 6% polyacrylamide gels containing 25 µM Mn^2+^-Phos-tag-acrylamide, which retards the migration of phosphorylated proteins [8]. Immunoblotting of these gels showed that a 20 minute treatment of the follicles with LH reduced the amount of the more slowly migrating species of NPR2, which is consistent with dephosphorylation.

## Conclusion

LH signaling rapidly reduces cGMP synthesis in the granulosa cells of the ovarian follicle, by dephosphorylating the NPR2 guanylyl cyclase. The resulting decrease in cGMP propagates through gap junctions to reduce cGMP in the oocyte, where it promotes the resumption of meiosis.
